# *De Novo* Transcriptome Sequencing Reveals Important Molecular Networks and Metabolic Pathways of the Plant, *Chlorophytum borivilianum*

**DOI:** 10.1371/journal.pone.0083336

**Published:** 2013-12-23

**Authors:** Shikha Kalra, Bhanwar Lal Puniya, Deepika Kulshreshtha, Sunil Kumar, Jagdeep Kaur, Srinivasan Ramachandran, Kashmir Singh

**Affiliations:** 1 Department of Biotechnology, Panjab University, Chandigarh, India; 2 G N Ramachandran Knowledge Center, Institute of Genomics and Integrative Biology (Council of Scientific and Industrial Research), New Delhi, India; Centro de Investigación y de Estudios Avanzados del IPN, Mexico

## Abstract

*Chlorophytum borivilianum*, an endangered medicinal plant species is highly recognized for its aphrodisiac properties provided by saponins present in the plant. The transcriptome information of this species is limited and only few hundred expressed sequence tags (ESTs) are available in the public databases. To gain molecular insight of this plant, high throughput transcriptome sequencing of leaf RNA was carried out using Illumina's HiSeq 2000 sequencing platform. A total of 22,161,444 single end reads were retrieved after quality filtering. Available (e.g., De-Bruijn/Eulerian graph) and in-house developed bioinformatics tools were used for assembly and annotation of transcriptome. A total of 101,141 assembled transcripts were obtained, with coverage size of 22.42 Mb and average length of 221 bp. Guanine-cytosine (GC) content was found to be 44%. Bioinformatics analysis, using non-redundant proteins, gene ontology (GO), enzyme commission (EC) and kyoto encyclopedia of genes and genomes (KEGG) databases, extracted all the known enzymes involved in saponin and flavonoid biosynthesis. Few genes of the alkaloid biosynthesis, along with anticancer and plant defense genes, were also discovered. Additionally, several cytochrome P450 (CYP450) and glycosyltransferase unique sequences were also found. We identified simple sequence repeat motifs in transcripts with an abundance of di-nucleotide simple sequence repeat (SSR; 43.1%) markers. Large scale expression profiling through Reads per Kilobase per Million mapped reads (RPKM) showed major genes involved in different metabolic pathways of the plant. Genes, expressed sequence tags (ESTs) and unique sequences from this study provide an important resource for the scientific community, interested in the molecular genetics and functional genomics of *C. borivilianum*.

## Introduction

*Chlorophytum borivilianum* is an important species of liliaceae family due to its exceptional medicinal properties. Overexploitation and extensive harvesting of the wild strands has threatened its status as ‘endangered’ species by International Union for Conservation of Nature and Natural Resources (IUCN) [1]. Due to its high medicinal properties, the species has been recognized as 26^th^ among the top priority medicinal plants to be protected and promoted by the Medicinal Plant Board, Government of India.

*Chlorophytum borivilianum* brag the exuberant references in many Ayurvedic classics like Charka Samhita (2^nd^ century B.C.), Sushrut Samhita (2^nd^ century A.D.), Raja Nighantu (17^th^ century A.D.) etc. (http://www.safedmusli.net). Its tubers are used for aphrodisiac, adaptogen, antiageing, health restorative and health promoting purposes. The amalgamation of *C. borivilianum* leaves with other herbs such as *Withania sominifera*, *Emblica officinalis* etc. makes the body resistant against sex related diseases and also delays menopause [2]. The above attributes have made *C. borivilianum* an essential ingredient in Ayurvedic, Unani and Allopathic formulations. Major phytochemical components reported from the roots of *C. borivilianum* include steroidal saponins, fructans and fructoligosaccharides (FOS), acetylated mannans, phenolic compounds and proteins [3], [4]. Steroidal saponins, are considered to be the principal bioactive components responsible for the pharmacological properties [5] and borivilianosides, furostane type steroidal saponins, have been isolated and characterized from this plant [6], [7].

Steroidal saponins are synthesized via the mevalonic acid (MVA) pathway, pervasively operating in cytoplasm [8], or through the newly discovered non-mevalonate pathway (MEP) located in plastids [9], [10]. Cyclization of precursor compound, 2, 3-oxidosqualene, involving oxidosqualene cyclase (OSC) combined with modifications on steroid skeletons like hydroxylations and glycosylations lead to the formation of various saponins. Several OSC genes like *cycloartenol synthase* (*CAS*), *lupeol synthase* (*LS*), *β- amyrin synthase* (*β- AS*) have been cloned from various plant systems [11], [12]. According to the proposed pathway [13], some specific CYP450s and UDP-glycosyltransferases (UGTs) may catalyze the conversion of cycloartenol to various steroidal saponins. Little is known about the molecular mechanism of the biosynthetic pathway downstream of cyclization process involved in saponin biosynthesis. The functional genomics studies in *C. borivilianum* is still in its infancy and few expressed sequence tags (ESTs) have been generated by our group [13], with an aim to identify the differentially expressed genes in the leaf and root tissues of *C. borivilianum*. Also, efforts are being made to clone the genes of interest via the candidate gene approach [14] for this plant.

ESTs play significant role in accelerating gene discovery, especially in non model organisms where reference genome sequence is unavailable, as it is a rapid and relatively economical method for analyzing the transcribed region of the genome [15]. Besides this, ESTs are also helpful in large scale expression analysis, improving genome annotation, identifying splice variants, molecular markers identification and physical mapping [16]. For large scale transcriptome analysis, next generation sequencing has evolved to be a very useful technique for providing large expression data in much shorter time period, depth and coverage to expedite understanding of metabolic pathway as well as contribute to comparative transcriptomics, evolutionary genomics and gene discovery [17], [18]. Illumina high throughput sequencing technology yields huge amount of parallel sequence short reads with larger coverage. Assembling these short read sequences is a challenging task in the absence of any reference sequence. However, many *de novo* assembly tools have been developed that can be used to analyze the short read sequences [19], [20].

We have undertaken the first global analysis of *C. borivilianum* transcriptome. Strategy has been developed for *de novo* assembly of transcriptome using short-read sequence data generated by Illumina RNA-Seq method in lieu to identify candidate genes involved in saponin biosynthetic pathway. Furthermore, GC content analysis, identification of EST-SSRs and gene expression analysis has also been done. Transcriptome coverage, at 22.42 megabase pairs, was comprehensive enough to discover all known genes of several major metabolic pathways. The data has been submitted to the Sequence Read Archive (SRA) of NCBI database under the accession ID PRJNA 196968 and will serve as a public information platform for further studies in *C. borivilianum*.

## Materials and Methods

### 2.1 Plant Material, growth conditions and saponins extraction

Plants of *C. borivilianum* were grown under controlled conditions in the growth chamber with a day/night temperature of 27±1°C, for 16 h photoperiod (flux density of 200 µmol m^−2^ s^−1^) with 30% relative humidity at Panjab University, Chandigarh (India). Plants were regenerated from the previous year's roots in the month of April. Young leaves and roots of a two month old plant were harvested during the period of active growth (June, average outside temperature 38°C), snap frozen in liquid nitrogen and later transferred to a −80°C freezer until further processing.

Total saponins were isolated from leaf and root tissues by soxhelation method and analyzed by thin layer chromatography (TLC) [21]. Briefly, powdered tissues were defatted with petroleum ether (60–80°C). Ethanolic extracts were prepared by extracting defatted tissues with 95% ethanol in a soxhlet extractor. Ethanolic extract was suspended in water (100 ml) and then extracted with n-butanol (300 ml). The volume of n-butanol soluble portion was reduced to half under reduced pressure and finally saponins were precipitated by addition of diethyl ether. Chromatographic analysis of ethanolic extract was performed on silica gel-GF254 precoated plates using chloroform: glacial acetic acid: methanol: water (16∶8∶3∶2, v/v) as mobile phase. Anisaldehyde (0.5 ml) mixed with glacial acetic acid (10 ml), methanol (85 ml) and concentrated sulphuric acid (5 ml) was used as spraying reagent. TLC plates, after spraying the reagent, were heated at 100°C and saponin spots were visualized.

### 2.2 RNA isolation, cDNA library construction and Illumina sequencing

Total RNA was extracted by combining the methods as described by Ghawana *et al* (2011), [22] followed by RNA purification and on column DNaseI digestion using miRNA Easy kit (Qiagen, Germany). The integrity of RNA samples was assessed by Agilent 2100 Bioanalyzer (Agilent Technologies, USA). Sample with RNA integrity number (RIN) value more than 8.0 was selected for further use.

Library construction and sequencing was performed at Microarray core facility, Huntsman Cancer Institute, University of Utah, Salt Lake City, Utah, USA. cDNA library was generated using TruSeq™ RNA Sample preparation kit (Illumina, USA) according to the manufacturer's instructions. Briefly, oligo(dT) beads were used to purify poly(A) mRNA from total RNA. mRNA was fragmented using RNA fragmentation kit (Ambion, USA). First strand cDNA was synthesized from the fragmented mRNA using random hexamer primer and reverse transcriptase (Invitrogen, USA). Single-end cDNA library was prepared in accordance with Illumina's protocol with an insert size of 150 bp. 50-cycled single end library sequencing was performed using Illumina Hiseq2000 platform. Raw reads were filtered to obtain high-quality clean reads by removing adaptor sequences, duplication sequences using FastQC software and bases with Phred score <20 were trimmed. Based on the quality check for each base pair in the reads, last two base pairs from each read were removed in order to minimize the sequencing error, which is usually higher in the 3′ end of reads.

### 2.3 *De novo* assembly of the sequences and clustering

All the assemblies were performed on Red Hat based SGI workstation with 48 cores, 2.27 GHz Intel Xeon processor and 50 Gb random access memory. We used SOAPdenovo (version 1.04; http://soap.genomics.org.cn/soapdenovo.html) which applies de Bruijn graph algorithm for *de novo* assembly of high quality (HQ) sequence reads to generate a non-redundant set of transcripts. Clean reads were first split into different ‘k-mers’ for assembly, in order to produce contigs, using the de Bruijn graph. K-mer size of 23 achieved best balance between the number of contigs produced, coverage and average sequence length obtained.

In order to reduce sequence redundancy, clustering process was supplemented with TIGR gene indices clustering tools (TGICL), which is based on pairwise sequence similarity and then assembly by individual clusters in order to retrieve better results [23]. Clusters were then passed to contig assembly program (CAP3) assembler for multiple alignments and consensus building. TGICL and CAP3 assembly were run under default parameters. Resulting singletons and consensus contigs were merged for final assembled transcripts.

### 2.4 Functional annotation and Classification

All the assembled unigenes (consensus and singletons) longer than 100 bp were annotated by assigning putative gene descriptions and Gene Ontology (GO) terms on the basis of sequence similarity with previously identified genes annotated with similar details. Unigenes were subjected to BLASTX search against non-redundant protein database and significant hits with e-value ≤1.0e^−05^ were extracted. Transcripts that did not show any significant hit were searched using BLASTN tool against the non-redundant database. Functional categorization by GO terms (http://www.geneontology.org) [24] and Enzyme Commission (EC) database was carried out based on Anno8r tool [http://www.nematodes.org/bioinformatics/annot8r/] with E-value threshold of 1.0e^−05^. GOSlim terms for molecular function, biological process, and cellular component categories associated with the best BLASTX hit with non- redundant database were assigned to the corresponding *C. borivilianum* transcripts. KOBAS 2.0 (http://kobas.cbi.pku.edu.cn/home.do) software was used for annotation of transcripts with KEGG pathways. For this, we first used KEGG orthology data provided at website and locally BLAST all transcripts with it. The transcripts showing similarity with KO database at e-value cut-off of 1e^−05^ were selected. The resultant data was used as an input for annotations wherein, we mapped transcript ID's to KO terms which finally annotate the transcripts with KEGG pathways. The ‘Identify’ program in KOBAS 2.0 uses annotation to identify enriched pathways. *Oryza sativa*, being a monocot, was used as background species. *Arabidopsis thaliana*, being a model organism was also used as background species. Hyper geometric distribution was used for p-value calculation. The false discovery rate (FDR) was calculated by using q-value and other parameters were used as default.

### 2.5 Read mapping onto C. borivilianum transcripts

The expression level of each assembled transcript was measured through reads per kilobase per million mapped reads (RPKM) values. All reads were mapped onto the non-redundant set of transcripts to quantify the abundance of assembled transcripts. SeqMap [25] was used for read mapping and rSeq [26] was then applied for RPKM based expression measurement. Assembled sequences were used as reference sequence to map back short reads and to measure RPKM for all assembled transcripts as suggested by Mortazavi et al. [26] and Jiang and Wong [25]. For RPKM measurement, filtered reads were first mapped back to various assembled transcripts and total mapped reads were estimated. Unique mapped reads were assigned to each assembled transcript allowing maximum two mismatches. For each such cluster having similar sequences in database, longest sequence was considered as the representative sequence for the unique gene it represented. The associated GO terms and Id's were parsed for each of such sequence and their corresponding RPKM values for different assembled genes were calculated.

### 2.6 GC content analysis and Simple Sequence Repeats (SSRs) identification

GC content analysis was done using in-house developed R script. A Perl script known as MIcroSAtellite (MISA, http://pgrc.ipk-gatersleben.de/misa/) was used to identify SSRs in the unigenes. Repeats of mono-nucleotide more than 10 times, di-nucleotides repeats more than 6 times, tri-, tetra-, penta- and hexa-nucleotide repeats more than 5 times were considered as search criteria in MISA script.

### 2.7 Identification of Transcription Factor families

For identification of transcription factor families represented in *C. borivilianum* transcriptome, transcripts were probed against all the transcription factor protein sequences at Plant transcription factor database (PlnTFDB; http://plntfdb.bio.uni-potsdam.de/v3.0/downloads.php) using BLASTX with an e-value cut-off of 1.0e^−05^.

## Results and Discussion

### 3.1 Transcriptome sequencing and *de novo* assembly

Illumina Hiseq 2000 (Illumina, USA) run representing the cDNA library from leaf tissues produced 22,595,634 single end (SE) reads. Each read was 50 bp in length encompassing nearly 4.0 Gb of sequencing data in fastq format. Since 3′ ends of reads are more prone to sequencing error, so after quality check for every 50 bp read, only 48 bases (excluding 2 bases at 3′ end) were considered for further analysis. Sequence data was filtered for low-quality reads and reads containing primer/adaptor sequences. Reads having the Phred quality score ≥20 (an error probability of 0.01) were selected for further use. After quality filtering, a total of 22,161,444 high-quality SE reads (3.8 Gb, 95% of the raw data) remained. Quality of the clean reads data was assessed using FASTX Toolkit (www.hannonlab.cshl.edu/fastxtoolkit).

**Figure 1:**
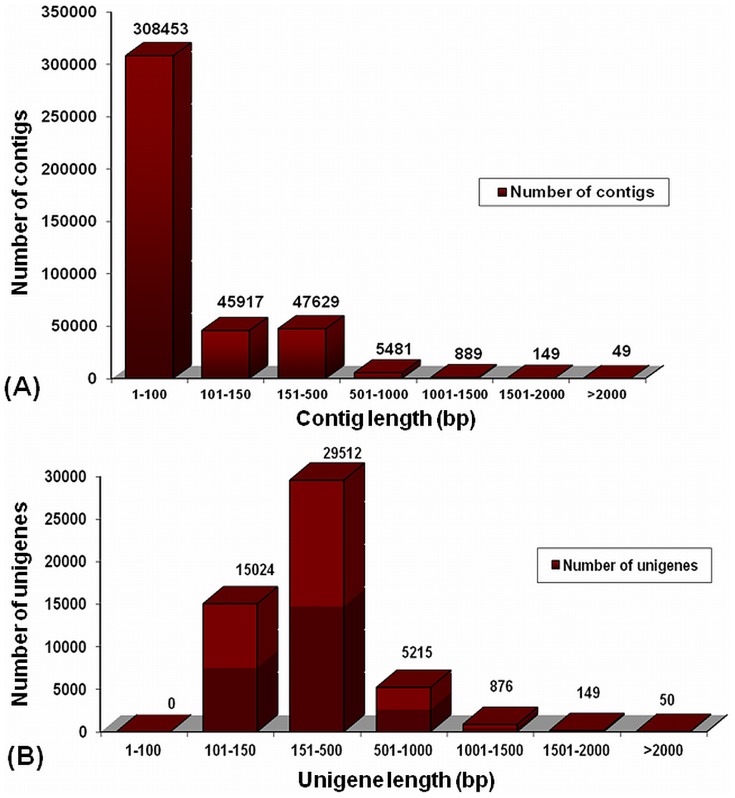
Overview of the *C. borivilianum* transcriptome assembly. (**A**) Size distribution of the contigs obtained from *de novo* assembly of high quality clean reads. (**B**) Size distribution of the unigenes produced from further assembly of contigs after clustering.

*De novo* assembly of *C. borivilianum* transcriptome was optimized after assessing effect of various k-mer lengths. The high quality trimmed reads (48 bp) were assembled using SOAPdenovo program at k-mer length of 17, 21, 23, 25, 27, 31, and 35 (Table 1). Total number of transcripts decreased linearly with the increment of k-mer size suggesting over-representation at lower k-mer and under-representation at higher k-mers. It was observed that sequences assembled at higher k-mer were enriched for transcripts with higher coverage/higher expression. For assembly process, only those reads were considered that produced high frequency k-mer. Several output parameters were analyzed that included total number of contigs, contigs with length 100 bp and above, N50 length, longest contig length, and average contig length as a function of k-mer. For the above mentioned data set, k-mer size of 23 emerged as the best for assembly with N50 length of 245 bp, largest contig length 3,168 bp and average contig length of 221 bp (Table 1). A total of 101,589 contigs having length of at least 100 bp were generated. These contigs made the final representatives for assembled sequences for this study. Total bases covered by contigs with length greater than 100 bp and above came out to be 22.47 Mb (Table 2). Although the majority of the contigs lie between 1 bp to 100 bp, a total of 6568 contigs with a size of 500 bp and above were are generated (Fig 1a).

**Table 1:** Effect of k-mer size on assembly of transcriptome data.

k-mer	Total contigs	Contigs >100 bp	Longest contig length (bp)	Average length (bp)	Total bases covered by 100 bp length	N50
17	1079131	99031	1862	178	17930159	180
21	516724	101445	3216	220	22401122	243
23	408567	101589	3168	221	2247281	245
25	337186	101765	3179	216	2207354	240
27	276611	101491	5256	211	2240112	232
31	183200	95717	5256	199	1911429	211

**Table 2:** Summary of filtered and assembled transcriptome data generated on Illumina Hiseq 2000 platform using RNA isolated from leaf tissue of *C. borivilianum.*

Total number of single-end reads	22,595,634
Number of reads obtained after quality filtering	22,161,444
Number of assembled transcripts	1,01,589
Average length of transcripts (bp)	221
Average coverage (bp)	22,472,81

### 3.2 Sequence clustering and similarity search

To reduce any redundancy, assembled sequences from the above analysis were clustered by hierarchical clustering with TGICL [27], which generated 1273 clusters. These clusters were further assembled using CAP3 program [28] and using 896 sequences, 448 consensus sequences were generated. This resulted in reduction of uniquely assembled contigs from 101,589 to 101,141. Contigs shorter than 100 bp were removed and total coverage with contig length ≥100 bp came out to be 22.42 Mb. Although majority of the contigs lie between 150 bp to 500 bp, we obtained a total of 6,290 contigs with a size of >500 bp (Fig 1b).

**Figure 2:**
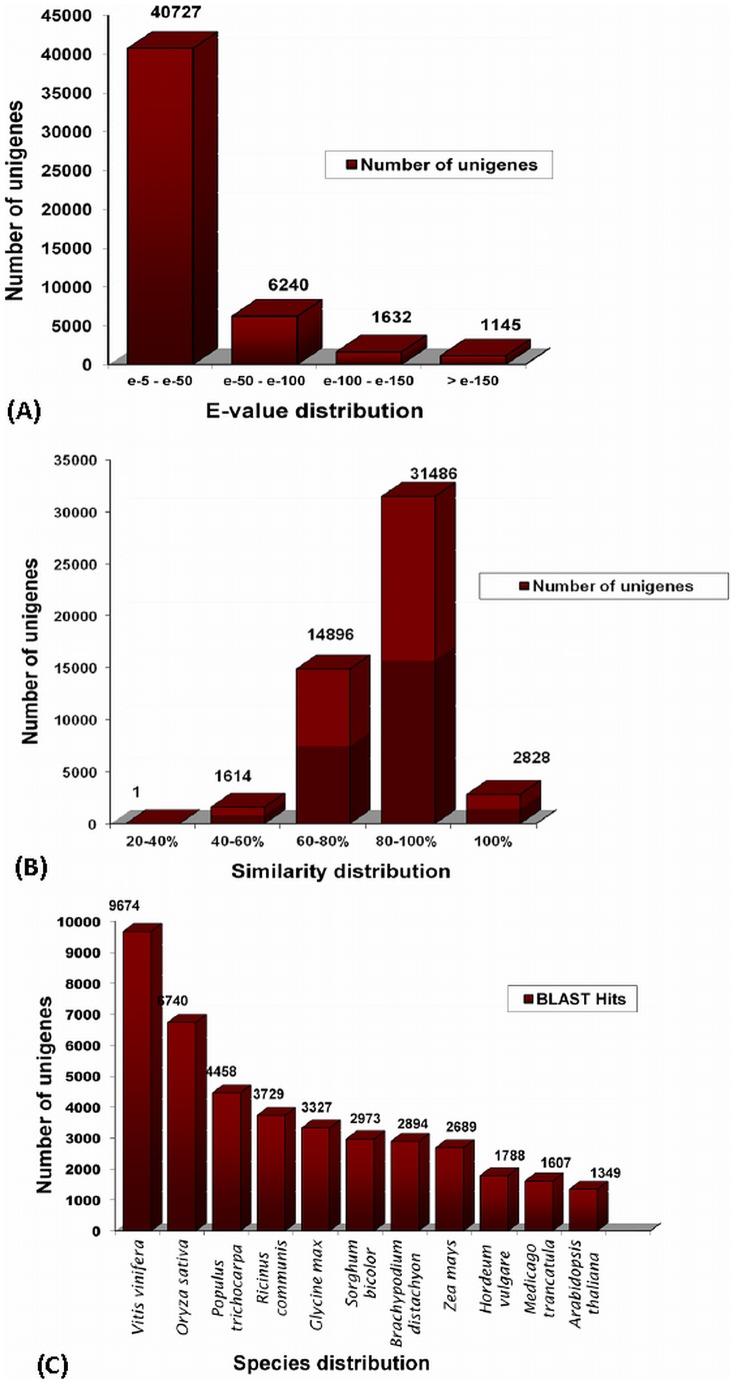
Similarity search analysis of unigenes against the NR database. (**A**) E-value distribution of the top BLAST hits for each unigene (E-value of 1.0e^−5^). (**B**) Similarity distribution of the best BLAST hits for each unigene. (**C**) Species distribution is shown as the percentage of the total homologous sequences (with an E-value ≤1.0e^−5^). We used the NCBI NR proteins database for similarity search and extracted the best hit of each sequence for analysis.

For contig annotation, similarity search was performed using BLASTX against the non-redundant (NR) protein database with an E-value cut off 1.0e^−05^. For read assembled transcript sequences, significant BLASTX hits were found for a total of 50,826 sequences (50.4% of the total unigenes obtained; Table S1). The E-value distribution of the top hits in the NR database showed that 15.9% of the mapped sequences have high similarity (<1.0e^−50^), while the other 84.1% of sequences, similarity ranged between 1.0e^−05^ to 1.0e^−50^ (Fig 2a). The similarity distribution revealed 67.5% of the query sequences have a similarity higher than 80%, while 32.4% of the hits have a similarity ranging from 20% to 80% (Fig 2b). Homologous genes come from several species, with 23.5% of the unigenes having the highest homology to genes from *Vitis vinifera* followed by *Oryza sativa* (16.3%), *Populus trichocarpa* (10.8%), *Ricinus communis* (9.0%), *Glycine max* (8.1%) and *Sorghum bicolor* (7.2%) (Fig 2c).

Due to lack of *C. borivilianum* genome information, nearly half of the unigenes did not show a match in the plant protein dataset of NR database. Moreover, 63.4% of the above mentioned 50,826 annotation descriptions were uninformative (e.g., ‘unknown’, ‘unnamed’, ‘putative’, or ‘hypothetical’ protein).

### 3.3 Validation of assembled sequences against the available ESTs of *C. borivilianum*

The assembled sequences were validated by sequence alignments against ESTs of *C. borivilianum* submitted at NCBI dbEST by our group [13]. BLASTN analysis of assembled transcripts was performed with 459 ESTs with an E-value threshold of 1.0e^−05^ (Table S2). Alignment parameters were manually checked and also analyzed for mis-assemblies. Significant hits were observed for 394 sequences (85.8%), while no hit could be obtained for 65 ESTs from the assembled transcript set. It was observed that most of the assembled transcript sequences aligned correctly and in continuous form, with average identity of 95.67%, showing good assembly quality. The possible reason for unmapped unigenes of *C*. *borivilianum* could be due the presence of relatively short and low quality singletons, UTR sequences far from the translation start or stop sites (>1,000 bp), fusion transcripts and those having incomplete coverage by the genome. Similar results were shown in *A. thaliana* where around 13% of the ESTs could not be aligned to the predicted genes [29] and in human only 64% of the reads could be mapped to the RefSeq database of well annotated human genes [30]. *Picorrhiza kurrooa* had 14% of the ESTs that could not be aligned to its predicted genes [31].

### 3.4 Functional annotation and classification of *C. borivilianum* transcriptome

**Figure 3:**
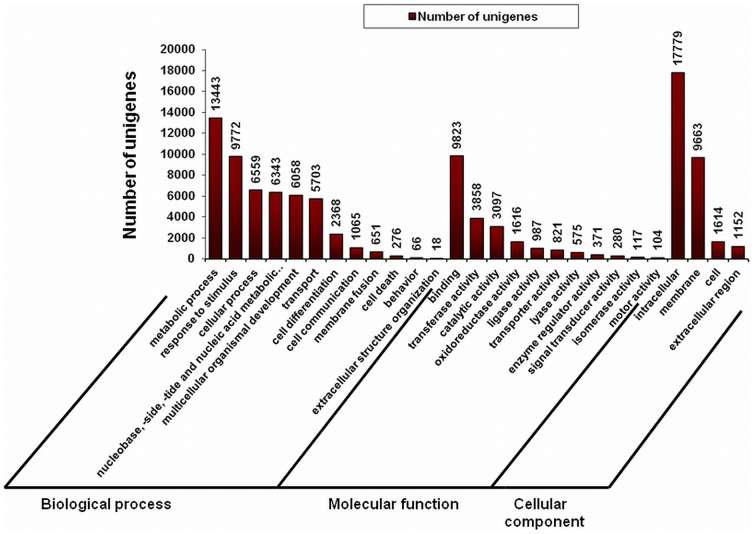
Gene Ontology (GO) classification of the *C. borivilianum* transcriptome. GO term assignments to *C. borivilianum* unigenes based on significant plant species hits against the NR database. Results are summarized into three main GO categories (biological process, cellular component, molecular function) and 27 sub-categories.

*Chlorophytum borivilianum* transcripts were assigned GO terms to describe functions of genes and associated gene products into three major categories namely, biological process, molecular function, and cellular component, and their sub-categories [24]. A total of 30,625 out of 50,826 assembled sequences yielded significant annotation against GO database. These genes were further classified into three major categories namely, biological process, molecular function, and cellular component using plant specific GO slims that broadly provides an overview of the ontology content. Functional classification of *C. borivilianum* transcripts in biological process category (Fig 3) showed that metabolic process (GO: 0008152), response to stimulus (GO: 0050896), nucleobase containing compound metabolic process (GO: 0006139) and extracellular structural organization (GO: 0009987) were among the highly represented groups indicating that the plant is undergoing rapid growth and extensive metabolic activity. In cellular component group, sequences related to the intracellular regions (GO: 0005622) and membrane regions were well-represented categories (GO: 0016020) (Fig 3). Transcripts belonging to major subgroups of molecular function category included binding functions (GO: 0005488), transferase activity (GO: 0016740), catalytic activity (GO: 0003824) and oxidoreductase activity (GO: 001649) (Fig 3). These GO annotations provide a comprehensive information on *C. borivilianum* expressed genes that are encoding diverse proteins.

**Figure 4:**
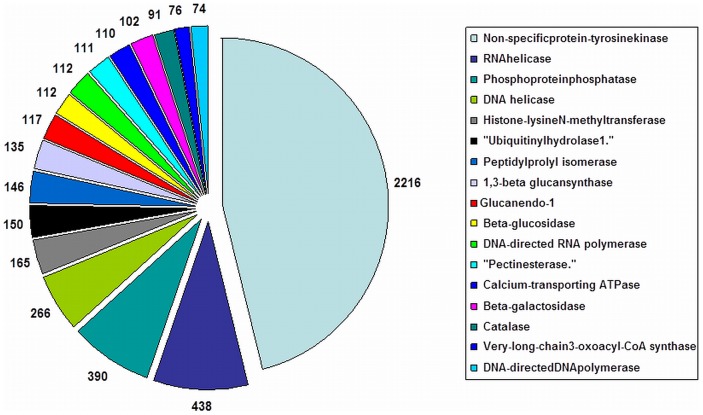
Functional characterization and abundance of *C. borivilianum* transcriptome for enzyme classes. *C. borivilianum* transcripts were classified in top 20 abundant enzyme classes; area under each pie represents the value in actual number of transcripts.

Best EC classification was obtained for 14,998 assembled sequences, which were annotated with 15,866 enzyme codes. Fig 4 lists major 20 abundant enzyme classes observed for *C. borivilianum* transcriptome. Interestingly, a large amount of assembled transcripts belonged to non-specific protein tyrosine kinases enzyme class alone (14.7%). In arabidopsis, this enzyme controls shoot and floral meristem size [32] and also contributes to signal transduction [33]. Presence of this enzyme in such abundance may suggest that the plant is in active metabolic phase.

**Figure 5:**
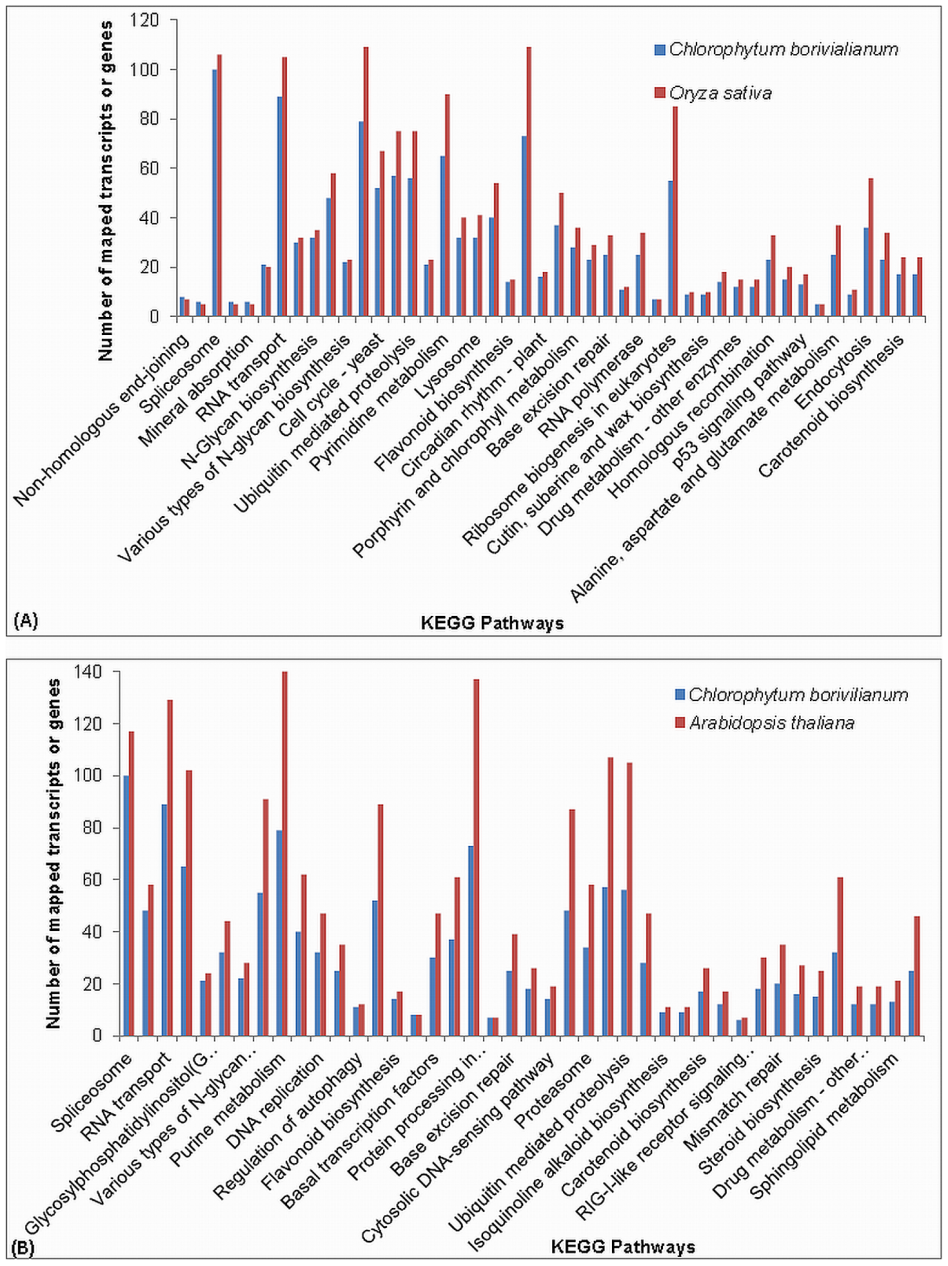
Overrepresented pathways in KEGG database with respect to A) *O. sativa* and B) *A. thaliana*.

We also compared KEGG pathways mapping of *C. borivilianum* with KEGG mapping of *O. sativa* and *A. thaliana*. Sample score corresponds to the transcripts of *C. borivilianum* mapped on KEGG pathways and background score is the total genes in the genome of the background species mapped on KEGG pathways. We found 50,034 transcripts showing similarity with GO database at e-value cut-off of 1e^−05^. These transcripts were used for annotation of KEGG pathways. These transcripts were annotated with 257 KEGG pathways which were present in *O. sativa* and *A. thaliana*. A total of 236 pathways were common in both of background species. 10 unique pathways of *O. sativa* and 11 of *A. thaliana* were also mapped. Total 3,805 transcripts were mapped on these KEGG pathways common with *O. sativa* and 3,806 transcripts were mapped on KEGG pathways common with *A. thaliana*. Out of these 43 KEGG pathways were significantly enriched (p-Value <0.05) with the genes present in *O. sativa.* Non-homologous end joining, Lipopolysaccharise biosynthesis, Spliceosome, RIG-I like receptor signaling pathway, mineral absorption, GPI-anchor biosynthesis, RNA transport, Basal transcription factors, N-Glycan biosynthesis and RNA degradation are top enriched pathways. In comparison to *A. thaliana*, 42 pathways were identified as enriched (p-value <0.05). Spliceosome, RNA degradation, RNA transport, Pyrimidine metabolism and GPI anchor biosynthesis were found to be most enriched pathways in comparison with *A. thaliana*. A total of 100 transcripts in *C. borivilianum* were mapped on spliceosome KEGG pathway compared to 106 genes of *O. sativa* and 117 genes of *A. thaliana.* Highest number transcripts (106) were mapped to Ribosome pathways in *C. borivilianum* compared to 257 genes of *O. sativa* and 311 genes of *A. thaliana*. Other pathways with highest transcripts mapping include RNA transport (89), Purine metabolism (79) and protein processing in endoplasm (73), thus suggesting that the plant was in actively growing stage at the time of harvesting. Besides all these pathways, flavonoid biosynthesis and steroid biosynthesis were the most enriched pathways in *C. borivilianum*. The enrichment of these pathways clearly suggested that the plant possess antioxidant properties, anti-arthritic properties, and anticancer properties. Pathways comparison (Fisher's Exact P-value <0.05) with respect to the background species *O. sativa* and *A. thaliana* is shown in Fig 5a and 5b. All the data of pathways mapping is provided in Table S3A and Table S3B for *O. sativa* and *A. thaliana* respectively.

### 3.5 Discovery of the transcripts encoding putative transcription factors

**Figure 6:**
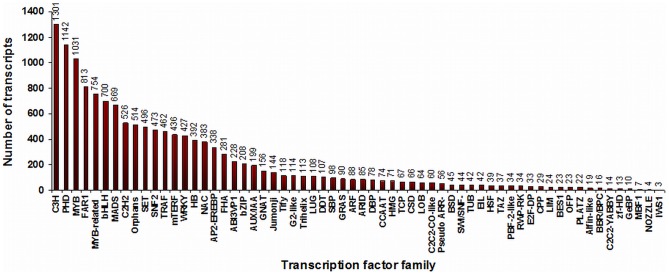
Distribution of *C. borivilianum* transcripts in different transcription factor families.

Transcription factors interact with the promoter regions of a gene and modulate its expression. Associative modulation of several plant processes suggests involvement of transcription factors (TFs) for coordinated regulation of gene expression. By sequence comparison with known transcription factor gene families, 8,369 putative *C. borivilianum* transcription factor genes, distributed in at least 62 families, were identified representing 8.3% of *C. borivilianum* transcripts (Fig 6). These genes covered transcription factor gene families like *MYB, MADS, Orphans, basic Helix-Loop-Helix (bHLH), bZIP, WRKY* and many more. Among all these TF gene families, *C3H, PHD* and *MYB* were most abundant families (Table S4). MYB family proteins are characterized by DNA-binding domains and it is the largest transcription factor family in *Arabidopsis thaliana* as well, where it comprises of 163 genes [34]. These TFs have been associated with varied processes. It has been observed that many protein members of the MYB, bZIP and WRKY transcription factor families insinuates the regulation of stress responses [35]. Members of C3H family are involved in embryogenesis [36] and members of PHD family TFs are involved in vernalization processes [37]. bHLH members are involved in controlling cell proliferation and development of specific cell lineages [38] and bZIP regulates processes including pathogen defense light and stress signaling seed maturation and flower development. A complete picture about evolution of various transcription factor families will emerge once the complete genome sequence of *C. borivilianum* will be available.

### 3.6 GC content and SSRs analysis from the transcriptome of *C. borivilianum*

Deep sequencing of *C. borivilianum* transcriptome rendered us with an opportunity to calculate the ratio of GC contents. Knowledge of GC content helps in understanding ecology and evolution of particular taxa. It also plays a vital role in gene and genome regulation and also helps in determining the physical properties of the genome. It is an important indicator of stability of DNA [39]. Average GC content of *C. borivilianum* transcripts was 44% (Figure S1). This is in range with the GC levels of coding sequences of *O. sativa* (43.6%) which also belong to monocot family.

Assembly of *C. borivilianum* was further assessed for the molecular markers. Morphological as well as biochemical markers are used in the authentication of herbal drugs. SSRs or microsatellite markers are polymorphic stretches of 1 to 6 nucleotide units repeated in tandem and randomly spread in eukaryotic genomes. SSRs are generally associated with functional and phenotypic variations. Moreover, SSRs allow identification of many alleles at a single locus, are easy to develop, distributed evenly all over the genome and are co-dominant [40]. SSRs are particularly useful when genome information of the crop is lacking. Since *C. borivilianum* is a cross pollinated species [41], and hence seed raised population will have variability.

For identification of SSRs, transcripts of *C. borivilianum* were analyzed with perl script MISA. 3,487 SSRs were identified in 3,321 (3.28%) transcripts comprehensively out of which, 160 sequences contained more than 1 SSR. With a frequency of over 43.1% (1,503/3,321), di-nucleotides were most abundant of all the SSRs obtained. Tri-nucleotides were more than 32.89% (1,147/3,487), followed by tetra-nucleotides (0.4%, 15/3,487) and penta-nucleotides (0.05%, 2/3487). Most prevalent of mononucleotide was poly-A. The edge of poly-A mono-nucleotide SSRs over others may be due to the presence of poly-A tails in the RNA sequences (Table S5). Di-nucleotide SSRs were the most abundant in the transcripts, which is similar to results obtained from other plants [42]. Among the di-nucleotide repeat classes, AG/GA/CT/TC (90.7%) was the most frequent dimer motif. Other dimer motifs included TG/CA and AT/TA (Table S5). CG repeats were infrequent in the plant (0.6%), which is consistent with previous observations [42], [43,]. Among tri-nucleotide repeats, CTC/CCT/GAG/GAA was the largest repeat class followed by CTT/AAG/GGA/AGA (Table 3). These findings indicated that unique sequences containing SSR markers were indeed abundant in *C. borivilianum*. Di- and tri-nucleotide SSR motifs and the number of repeats are presented in Table 3. In particular, several SSR motifs were linked with the unique sequences encoding enzymes (e.g. MVK, DXS, SqS) involved in saponin biosynthesis (Table 4). These unique sequence-derived markers generated in this study represent a valuable genetic resource for future studies in this and related species.

**Table 3:** Simple sequence repeats (SSRs) identified in transcripts of *C. borivilianum.*

SSR mining	Number
Total number of sequences examined:	10,11,41
Total size of examined sequences (Mb):	22.42
Total number of identified SSRs:	3,487
Number of SSR containing sequences:	3,321 (3.28%)
Number of sequences containing more than one SSR:	160
Number of SSRs present in compound formation:	109

Distribution of SSRs in different repeat types Unit size	Number of SSRs (%)
Mononucleotide	820 (23.42%)
Dinucleotide	1,503 (43.1%)
Trinucleotide	1,147 (32.89%)
Tetranucleotide	15 (0.4%)
Pentanucleotide	2 (0.05%)

**Table 4:** The discovery of SSR motifs in putative genes involved in saponin biosynthesis.

Gene name	Unique sequence	SSR motif	Number of repeats	SSR start (bp)	SSR end (bp)	Sequence length (bp)
*Mevalonate kinase (MVK)*	contig 780100	ag	7	6	19	1326
1-*deoxy-D-xylulose 5-phosphate synthase* (*DXS*)	contig 801760	ac	20	1	20	787
*DXS*	contig 809736	ta	16	64	79	1647
*Squalene synthase (Sqs)*	contig 676689	cat	16	1	16	355

### 3.7 Analysis of metabolic pathway genes

Saponins present in *C. borivilianum* are the primary source of its significant medicinal properties and are synthesized by mevalonate and non-mevalonate pathways in plants. TLC analysis indicated that significant levels of saponins were present in *C. borivilianum* tissues (Figure S2). Nearly 10 spots were visualized on TLC plate upon derivatization with anisaldehyde - sulfuric acid reagent. Previous studies reported that initial reactions of isoprenoid biosynthetic pathway occur in leaves, while later step modifications and storage of saponins occurs in roots [13], [14], thus the amount of saponins is high in roots. There are also few reports on the presence of flavonoid compounds in *C. borivilianum* [44]. These reports very well validate the presence of saponins and flavonoids in *C. borivilianum*, thus, our interest was to identify the genes responsible for biosynthesis of saponins and other metabolites in our dataset. The genes related to following pathways/compounds were identified:

#### 3.7.1. Saponin biosynthesis pathway

**Figure 7:**
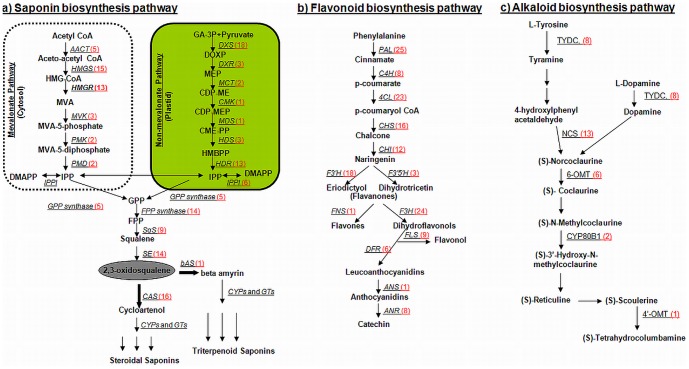
*Chlorophytum borivilianum* unigenes involved in two secondary metabolic pathways. *C. borivilianum* unigenes involved in; (A) saponin biosynthesis, (B) flavonoid biosynthesis and (C) alkaloid biosynthesis. Red number in the bracket following each gene name indicates the number of corresponding unigenes.

Saponins are derived from geranyl pyrophosphate (GPP). GPP is synthesized by sequential head to tail addition of isopentenyl pyrophosphate (IPP) and its allelic isomer dimethylallyl pyrophosphate (DMAPP) [45]. IPP and DMAPP are synthesized via the cross talks between the cytosolic mevalonate (MVA) pathway and plastidial 2-C-methyl-D-erythritol-4-phosphate (MEP) pathway [46]. MVA pathway starts from the condensation of acetyl-CoA [47], whereas MEP pathway needs pyruvate and _lyceraldehydes 3-phosphate [48]. In *C. borivilianum* transcriptome dataset, multiple transcripts encoding almost all known enzymes involved in the MVA pathway, MEP pathway and saponin biosynthesis pathway were identified (Fig 7a). In almost all the cases, more than one unique sequence was annotated as same enzyme. These unique sequences may either represent different fragments of a single transcript or different members of a gene or both. MVA pathway starts with the condensation of three molecules of acetyl-CoA while in methylerythritol phosphate (non-mevalonate) pathway, isopentenyl diphosphate (IPP) is formed from pyruvate and _lyceraldehydes-3-phosphate. Both biosynthetic routes yield the activated isoprene units, dimethylallyl diphosphate (DMAPP) and IPP [49]. Genes involved in MVA pathway that were found in our transcriptome included *acetyl CoA- acetyltransferase* (EC 2.3.1.9, 5 unigenes), *HMG-CoA reductase* (EC 1.1.1.34, 13 unigenes), *HMG-CoA synthase* (EC 2.3.3.10, 15 unigenes), *mevalonate kinase* (EC 2.7.1.36, 3 unigenes), *phosphomevalonate kinase* (EC 2.7.4.2, 2 unigenes), *mevalonate diphosphate decarboxylase* (EC 4.1.1.33, 2 unigenes). MEP pathway genes are also converted to IPP by reaction catalyzed by series of enzymes. These included *1*-*deoxy-D-xylulose-5-phosphate synthase* (EC 2.2.1.7, 18 unigenes), *1-deoxy-D-xylulose-5-phosphate reductoisomerase* (EC 1.1.1.267, 3 unigenes), *2-C-methyl-D-erythritol 4-phosphate cytidylyl transferase* (EC 2.7.7.60, 2 unigenes), *4-diphosphocytidyl-2-C-methyl-D-erythritol kinase* (EC 2.7.1.148, 1 unigene), *2-C-methyl-D-erythritol, 2,4-cyclodiphosphate synthase* (EC 4.6.1.12, 1 unigene), *4-hydroxy-3-methylbut-2-enyl diphosphate synthase* (EC 1.17.7.1, 3 unigenes), *4-hydroxy-3-methylbut-2-enyl diphosphate reductase* (EC 1.17.1.2, 13 unigenes). IPP was then converted to squalene by a series of enzymes which included *isopentenyl diphosphate isomerase* (EC 5.3.3.2, 6 unigenes), *geranylgeranyl diphosphate synthase* (EC 2.5.1.29, 5 unigenes) *farnesyl diphosphate synthase* (EC 2.5.1.10, 14 unigenes), *squalene synthase* (EC 2.5.1.21, 9 unigenes). Subsequently, *squalene monooxygenase* (EC 1.14.13.132, 14 unigenes) catalyzes the conversion of squalene to 2, 3-oxidosqualene. The cyclization event of 2, 3-oxidosqualene is catalyzed by a class of enzymes, OSCs. Cyclization of 2, 3-oxidosqualene is rate limiting step and this event is also the branch point for sterol and triterpenoid biosynthesis in many plants [50]. Three OSC genes of *C. borivilianum*, *cycloartenol synthase* (E.C. 5.4.99.8, 16 unigenes) [51], *β-amyrin synthase* (E.C. 5.4.99.39, 1 unigene) [52] and *Dammarenediol II synthase* (E.C. 4.2.1.125, 1 unigene) [53] exist in our dataset. Steroidal saponins, borivilianosides, are the major type of saponins present in *C. borivilianum* [6]. As yet, no reports reveal the presence of triterpenoid type of saponins in *C. borivilianum.* However, two singleton sequences (Transcript ID: 664097 and 798016) matched with *β-amyrin synthase* and *Dammarenediol II synthase* of *V. vinifera* and *R. communis* respectively.

#### 3.7.2 Flavonoid biosynthesis pathway

Genes associated with flavonoid biosynthesis pathway were also detected in the dataset (Fig 7b). Flavonoids are synthesized via the phenylpropanoid pathway and are converted from phenylalanine to chalcone by the enzymes *phenylalanine ammonia lyase* (EC 4.3.1.24, 25 unigenes), *cinnamate 4-hydroxylase* (EC 1.14.13.11, 8 unigenes), *4-coumarate CoA ligase* (EC 6.2.1.12, 23 unigenes), and *chalcone synthase* (EC 2.3.1.74, 16 unigenes). *Chalcone isomerase* (EC 5.5.1.6, 12 unigenes) then catalyzes the isomerisation of chalcones into naringenin. Naringenin can be converted by *flavonoid 3′- hydroxylase* (EC 1.14.13.21, 18 unigenes, 421.18) and *flavonoid 3′, 5′-hydroxylase* (EC 1.14.13.88, 3 unigenes) to produce eriodictyol and dihydrotricetin, respectively. *Flavone synthase* (EC 1.14.11.22, 1 unigene) catalyzes the conversion of flavanones to flavones, and *flavanone 3-hydroxylase* (EC 1.14.11.9, 24 unigenes) can convert these flavanones to dihydroflavonols. Dihydroflavonols can then lead to production of flavonols and flavan - 3, 4-diols (leucoanthocyanidin), reactions being catalysed by *flavonol synthase* (EC 1.14.11.23, 9 unigenes) and by *dihydroflavonol 4-reductase* (EC 1.1.1.219, 6 unigenes) repectively. Leucoanthocyanidins can be converted either to anthocyanidins and subsequently anthocyanins through the subsequent action of *anthocyanidin synthase* (EC 1.14.11.19, 6 unigene) or reduced to catechins through the action of the enzyme and *anthocyanidin reductase* (EC 1.3.1.77, 8 unigenes). Genes of the phenylpropanoid pathway have already been reported in several plant species such as *Camellia sinensis* [54], [55], *Petroselinum hortense* (Parsely) [56], *Zea mays* [57], *Arabidopsis thaliana* [58], *Vitis vinifera* [59], *Citrus unshiu* (Marc.) [60] and *Fragaria* spp. [61]. Above descriptions showed vertical pathway responsible for the formation and conversion of the sub-categories of flavonoids.

Another revelation came out with the genes involved in isoflavonoid metabolism. These genes included *2-hydroxyisoflavanone synthase* (EC 1.14.13.86, 3 unigenes), *isoflavone 4′-O-methyltransferase* (EC 2.1.1.46, 1 unigene), *isoflavone 7-O-methyltransferase* (EC 2.1.1.150, 2 unigenes*), isoflavone 2′-hydroxylase* (EC 1.14.13.89, 9 unigenes) and *isoflavone reductase* (EC 1.3.1.45, 12 unigenes). *C borivilianum* is not known to produce isoflavones. Similar kinds of findings have been reported in other non-isoflavone accumulating plants [62], suggesting homologs of *isoflavone reductase* and *isoflavone O-methyltransferase* may have general metabolic functions in many plant species.

#### 3.7.3. Discovery of transcripts encoding CYPs and UGTs involved putatively in saponin and flavonoid biosynthesis

Cytochrome P450 (CYP 450) are versatile biocatalysts. These enzymes constitute largest family of plant proteins (http://drnelson.utmem.edu/CytochromeP450.html). CYP450s are involved in the NADPH-dependent regio- and stereo-specific oxygenations during the biosynthesis of terpenoids, sterols, lignins, hormones, fatty acids, pigments, and phytoalexins in plants [63]. In biosynthesis of steroidal-type of saponins, CYP450 catalyzes the conversion of obtusifoliol to Δ^8, 14^ – sterol. A total of 490 unique transcripts, were annotated as CYP450s (Table S6). Previous studies reported characterization of *obtusifoliol 14-alpha-demethylase* (EC 1.14.13.70, 7 unigenes) of CYP51 family from *Sorghum bicolor* [64] and *Triticum aestivum* [65] and CYP 710 [66] from *Arabidopsis*, both of which are involved in steroid saponin biosynthesis. Therefore, the CYP450s belonging to CYP51 and CYP710 family might be involved in borivilianoside biosynthesis in *Chlorophytum* genus.

Amongst reactions for the modification of secondary metabolites, glycosylation plays an important role in plants, contributing to biosynthesis and storage of secondary metabolites [67]. *UDP-glycosyltransferases* (UGTs) catalyze the transfer of sugar residues from uridine diphosphate sugars to an acceptor. Several unigenes encoding for different type of glycosyltransferases were found in our dataset (Table S7). Out of these, UGTs supposed to be involved in saponin biosynthesis included *soyasaponin III rhamnosyltransferase* (EC 2.4.1.273, 4 unigenes) and *sterol 3- beta- glucosyltransferase* (EC 2.4.1.173, 9 unigenes). *Soyasaponin III rhamnosyltransferase* is involved in the biosynthesis of soyasaponin I in *Glycine max* [68]. The enzyme has strong sugar donor specificity for UDP-rhamnose. It does not show any activity with UDP-glucose, UDP-galactose or UDP- glucuronic acid. *Sterol 3- beta- glucosyltransferase* on the other hand is involved in the glycosylation activity of different type of sterols (e.g. sitosterol, stigmasterol etc.) present in the plant. This enzyme is thought to be involved in steroid metabolism.

UGTs involved in the glycosylation of flavonoids were also present in *C. borivilianum* transcriptome dataset, some of which were *anthocyanidin 3-O-glucosyltransferase* (EC 2.4.1.115, 16 unigenes), *anthocyanin 3′-O-beta-glucosyltransferase* (EC 2.4.1.238, 14 unigenes), and *flavonol-3-O-glucosyltransferase* (EC 2.4.1.91, 46 unigenes). Presence of such glycosylation enzymes and cytochrome P450 genes might contribute to extensive modifications of various secondary metabolites in *C. borivilianum*.

#### 3.7.4. Alkaloid biosynthesis pathway

Alkaloids are heterocyclic nitrogen compounds synthesized from amino acids and are the largest groups of natural plant products [69]. Potent biological activity of some alkaloids has led to their exploitation as pharmaceuticals, stimulants, narcotics and poisons. Plant-derived alkaloids currently in clinical use include, but not limited to, analgesics morphine and codeine, anticancer agents vinblastine and taxol, gout suppressant colchicine, muscle relaxant (C)-tubocurarine, antiarrythmic ajmaline, antibiotic sanguinarine, and sedative scopolamine. Other important alkaloids of plant origin include caffeine, nicotine, cocaine, and synthetic *O, O*-acetylated morphine derivative heroin.

In *C. borivilianum* transcriptome dataset, in addition to saponins and flavonoids, we also discovered genes involved in the biosynthesis of benzylisoquinoline alkaloids (BIA). Amino acid tyrosine is the precursor of benzylisoquinoline alkaloids. BIA biosynthesis begins with a metabolic lattice of decarboxylations, *ortho* hydroxylations and deaminations that convert tyrosine to both dopamine and 4- hydroxyphenylacetaldehyde [70]. This reaction is catalyzed by *tyrosine decarboxylase* (TYDC; EC 4.1.1.25, 8 unigenes), the only enzyme involved in these early steps that has been purified [71], and for which the corresponding cDNA has been cloned [72], [73] (Fig 7c). TYDC cDNAs have also been reported from parsley [74] and *Arabidopsis thaliana* [75], which do not accumulate tyrosine-derived alkaloids. TYDC mRNAs were shown to be rapidly induced in response to elicitor treatment [74], [75] and pathogen challenge [76] in various plants. Induction of TYDC mRNAs in parsley and *Arabidopsis* suggests that, in addition to BIAs, tyramine serves as precursor to a ubiquitous class of defense responsive metabolites. Dopamine and 4-hydroxyphenylacetaldehyde are condensed by *norcoclaurine synthase* (*NCS*) (EC 4.2.1.78, 13 unigenes) to yield the trihydroxybenzylisoquinoline alkaloid (*S*)- norcoclaurine, which is the central precursor to all BIAs in plants (Fig 7c) [77]. (*S*)-Norcoclaurine is converted to (*S*)-reticuline by a *6-O-methyltransferase* (EC 2.1.1.128, 6 unigenes), an *N-methyltransferase*, a P450 hydroxylase (EC 1.14.13.71, 2 unigenes) and a *4′-O-methyltransferase*. SAM-dependent *6-O-* and *4′-O-methyltransferases* (6-O-MT and 4′-O-MT, respectively) have been purified from cultured *Coptis japonica* cells [78], and the corresponding cDNAs were isolated and characterized [79]. (S)-reticuline is the central intermediate that can undergo various rearrangements and modifications to yield different structural classes of benzylisoquinolines. Berberine Bridge Enzyme (BBE) (*Reticuline oxidase*; EC 1.21.3.3, 1 unigene) that catalyses the conversion of reticuline to (S) - scoulerine was also found but we could not discover the enzymes that are involved beyond BBE in this pathway.

#### 3.7.5. Discovery of anticancer and plant defense genes

*C. borivilianum* possess antitumor and anticancer properties [80]. It contains cytotoxic steroidal glycoside saponinchloromaloside-A and spirostanolpentaglycosides embracing beta-D-apiofuranose which are used for their anticancer properties [81]. In the present study, for the first time, we reported the presence of genes involved in taxol biosynthesis. *3′-N-debenzoyal- 2′deoxytaxol N- benzoyaltransferase, taxane 10 - beta - hydroxylase* (*5-α-taxadienol-10-β-hydroxylase*) and *deacetyl baccatin* genes were discovered that are involved in taxol biosynthetic pathway. Few genes namely ± *neomenthol dehydrogenase* and *pulegone reductase,* involved in menthol biosynthesis pathway, were also discovered. Menthol biosynthetic genes are primarily involved in plant defense, although there are evidences that menthol has a potent anticancer property, effecting cell death through TRPM8 receptor [82]. Further studies on this pathway could be beneficial as the combination of *C. borivilianum* with other herbs could be beneficial for cancer treatment.

### 3.8 Quantification of *C. borivilianum* transcripts

All the reads were mapped onto the non-redundant set of *C. borivilianum* transcripts which revealed that the number of reads corresponding to each transcript ranged from 2 to 13289 with an average of 94 reads per transcript indicating a very wide range of expression in transcripts of *C. borivilianum* (Table S8). Minimum coverage (RPKM) of a *C. borivilianum* transcript was 3.38 and maximum of 4328.27 with an average of 55.78 (Figure S3). Low expressing transcripts were also present in our assembly. We also studied expression of various genes that were involved in the saponin biosynthesis and flavonoid biosynthesis pathways. We found that expression of unigene 807650 annotated as *Acetyl Co-acetyl transferase* (AACT), unigene662091 annotated as *HMG-CoA synthase*, unigene 757982 annotated as *HMG-CoA reductase* and unigene724460 annotated as *Squalene monooxygenase* was highest among saponin biosynthesis pathway genes (Table 4). AACT participates in 10 different metabolic pathways and HMG-CoA, synthesized by *HMG-CoA synthase* is an intermediate both in the saponin biosynthesis and in ketogenesis. This may be the reason of its high abundance in *C. borivilianum* transcriptome dataset. The expression of *cycloartenol synthase* (EC 5.4.99.8) (100 reads) was 5 fold higher than that of *β-amyrin synthase* (21 reads) (EC 5.4.99.39) (Table 5) thus, further confirming the diversion of the pathway towards the synthesis of steroidal type of saponins in this plant. Most of the genes of mevalonate pathway had expression greater than the genes of non-mevalonate pathway (Table 5). It might be due to the fact that mevalonate pathway is more active in the production of saponins in *C. borivilainum* as compared to non-mevalonate pathway as is the case commonly seen in monocot plants. In case of flavonoid biosynthesis, expression of *4-coumarate CoA ligase, dihydroflavonol 4-reductase, flavonol synthase, flavanone 3-hydroxylase* was the highest (Table 6).

**Table 5:** Identification of genes involved in saponin biosynthesis along with their RPKM values.

Gene Name	EC number	Transcript ID	Total ESTs	RPKM value	Number of reads
*acetyl Co-acetyl transferase*	2.3.1.9	contig 662605, 789282, 807132, 807650, 810900	5	420.22	2162
*HMG-CoA synthase*	2.3.3.10	contig 617027, 642120, 642122, 642652, 643560, 648291, 662091, 685089, 714060, 719432, 730642, 750334, 752180, 787594, 791328	15	588.8	399
*HMG-CoA reductase*	1.1.1.34	contig 616693, 642352, 728682, 731828, 739034, 750024, 751864, 757982, 761916, 765000, 777172, 789074, 805890	13	684	1144
*mevalonate kinase*	2.7.1.36	contig 780100, 790236, 808328	3	116.9	337
*phosphomevalonate kinase*	2.7.4.2	contig 796674, 802836	2	83.9	351
*mevalonate diphosphate decarboxylase*	4.1.1.33	contig 731872, 807394	2	183.9	932
*1-deoxy-D-xylulose-5-phosphate synthase*	2.2.1.7	contig 630630, 639174, 659061, 672215, 692780, 701402, 716282, 758550, 770832, 776522, 785368, 788176, 796714, 799960, 801760, 807536, 809736, 812228	18	33.8	31
*1-deoxy-D-xylulose-5-phosphate reductoisomerase*	1.1.1.267	contig 767146, 807128, 811584	3	49.4	325
*2-C-methyl-D-erythritol 4-phosphate cytidylyl transferase*	2.7.7.60	contig 697584, 812958	2	35.05	262
*4-diphosphocytidyl-2-C-methyl-D-erythritol kinase*	2.7.1.148	contig 815196	1	30.19	302
*2-C-methyl-D-erythritol, 2,4-cyclodiphosphate synthase*	4.6.1.12	contig 809428	1	35.16	200
*4-hydroxy-3-methylbut-2-enyl diphosphate synthase*	1.17.7.1	contig 768924, 813016, 815362	3	91.9	179
*4-hydroxy-3-methylbut-2-enyl diphosphate reductase*	1.17.1.2	contig 663803, 674257, 674259, 697268, 713070, 757110, 770076, 775264, 776012, 776014, 780586, 780710, 813560	13	91.6	217
*isopentenyl diphosphate isomerase*	5.3.3.2	contig 627116, 640576, 677655, 684005, 771148, 793800	6	239.49	128
*geranylgeranyl diphosphate synthase*	2.5.1.29	contig 669873, 709788, 771708, 791512, 804002	5	37.97	166
*farnesyl diphosphate synthase*	2.5.1.10	contig 622530, 663119, 669873, 694410, 709520, 709788, 759242, 764802, 771708, 774914, 782044, 785872, 791512, 804002	14	337.61	174
*squalene synthase*	2.5.1.21	contig 660543, 669241, 676689, 684225, 720378, 762052, 771110, 771878, 776032	9	178.12	119
*squalene monooxygenase*	1.14.13.132	contig 625956, 650031, 684205, 699388, 718888, 724460, 735738, 749834, 752160, 753330, 759096, 772472, 773000, 788034	14	789.79	897
*cycloartenol synthase*	5.4.99.8	contig 634716, 641806, 644535, 660567, 679937, 737170, 761218, 762950, 777784, 785022, 796606, 802302, 805366, 814320, 815892	16	177.58	100
*β-amyrin synthase*	5.4.99.39	contig 664097	1	30.56	21
*dammarenediol II synthase*	4.2.1.125	contig 663453	1	125.43	85

**Table 6:** Genes involved in flavonoid biosynthesis along with their RPKM values.

Gene Name	EC number	Transcript ID	Total ESTs	RPKM value	Number of read
*phenylalanine ammonia lyase*	4.3.1.24	contig 634698, 642066, 657575, 668909, 672281, 691416, 702626, 703366, 703960, 716568, 719604, 735030, 743228, 748710, 753296, 754648, 755468, 760124, 760936, 769576, 777484, 783272, 788948, 790736, 799910	25	241.09	474
*cinnamate 4-hydroxylase*	1.14.13.11	contig 626042, 673051, 688226, 704750, 755308, 758396, 791376, 794622	8	207.68	111
*4-coumarate CoA ligase*	6.2.1.12	contig 658459, 673103, 685642, 712208, 722868, 734074, 750294, 752608, 757946, 758914, 759276, 760860, 761416, 775342, 776318, 790346, 790364, 792818, 793014, 793670, 803146, 811102, 815080	23	1206.79	979
*chalcone synthase*	2.3.1.74	contig 619401, 623782, 642216, 664433, 694872, 703744, 711022, 729452, 742538, 750790, 752302, 775504, 776662 790158, 803096, 804748	16	158.11	83
*chalcone isomerase*	5.5.1.6	contig 618735, 654743, 666277, 716164, 716334, 761068, 765552, 772422, 776068, 783048, 785354, 790738	12	343.98	174
*flavonoid 3′- hydroxylase*	1.14.13.21	contig 645173, 688012, 697330, 716994, 717416, 719790, 732470, 733298, 746820, 756294, 765426, 780410, 781518, 796884, 799424, 805552, 813496	18	421.18	1013
*flavonoid 3′, 5′-hydroxylase*	1.14.13.88	contig 638078, 679673, 705666	3	31.43	18
*flavone synthase*	1.14.11.22	contig 682129	1	30.66	24
*flavanone 3-hydroxylase*	1.14.11.9	contig 617797, 622328, 628840, 631844, 649435, 649459, 656551, 665101, 712318, 715714, 721514, 724718, 728206, 741180, 760132, 763024, 767854, 789424, 790626, 792572, 800076, 801784, 803294, 812892	24	475.11	263
*flavonol synthase*	1.14.11.23	contig 631844, 656551, 721514, 741180, 763024, 767854, 800076, 801784, 812892	9	475.11	263
*dihydroflavonol 4-reductase*	1.1.1.219	contig 701984, 702106, 781542, 789140, 789820, 796570	6	729.12	679
*anthocyanidin synthase*	1.14.11.19	contig 698156, 706394, 733490, 752474, 778182, 794658	6	18.9	61
*anthocyanidin reductase*	1.3.1.77	contig 660955, 667861, 675719, 718414, 719766, 730968, 756916, 794760	8	22.83	17

## Conclusion

In conclusion, identification of genes involved in saponin and flavonoid biosynthesis will contribute to future functional studies in the plant and provide a basis for improving production levels in plants or in microbial hosts by metabolic engineering. In our data set, we also identified transcripts that encode for enzymes involved in anticancer and plant defense properties. Further, we annotated a large number of genes involved in the various pathways. Dataset of assembled *C. borivilianum* unigenes presented here will provide the foundation for other functional and comparative genomic studies.

## Supporting Information

Figure S1 **Guanine-cytosine (GC) content analysis of** ***C. borivilianum*** **transcripts. (TIF)**
(TIF)

Figure S2 **Analysis of total saponins contents in root and leaf tissue of** ***C borivilianum*** **by soxhelation method and TLC.**
(TIF)

Figure S3 **Distribution of RPKM values for different transcripts in** ***C. borivilianum*** **transcriptome.**
(TIF)

Table S1 **Assembled unigenes after CAP3 assembly.**
(XLS)

Table S2 **Assembly validation with known** ***C. borivilianum*** **leaf nucleotide sequences.**
(XLS)

Table S3 ***Chlorophytum borivilianum*** **metabolic pathway analysis using KEGG with; (A)** ***O. sativa*** **and (B)** ***A. thaliana*** **background databases.**
(XLS)

Table S4 **Details on transcription factor (TF) families identified in** ***C. borivilianum.***
(XLS)

Table S5 **Simple sequence repeats (SSRs) identified in transcripts of** ***C. borivilianum*** **.**
(XLS)

Table S6 **Annotated cytochrome P450s (CYPs) in** ***C. borivilianum*** **transcriptome.**
(XLS)

Table S7 **Annotated glycosyltransferases (GTs) in** ***C. borivilianum*** **transcriptome.**
(XLS)

Table S8 **Reads per exon kilobase per million (RPKM) based expression of annotated genes of** ***C. borivilianum*** **transcriptome. (XLS)**
(XLS)

## References

[1] Bordia PC, Joshi A, and Simlot MM (1995) Safed musli In: K.L. Chadha and R. Gupta ed. Advances in Horticulture Vol. 11 - Medicinal and Aromatic Plants. (Malhotra Publishing House, New Delhi) pp- 429–449.

[2] Kaushik N (2005) Saponins of *Chlorophytum* species. Phytochem Rev 4: 191–196.

[3] Narasimhan S, Govindarajan R, Madhavan V, Thakur M, Dixit VK, et al (2006) Action of (2→1)fructo-oligopolysaccharide fraction of *Chlorophytum borivilianum* against streptozotocin-induced oxidative stress. Planta Medica 72: 1421–1424. 17051465 10.1055/s-2006-951705

[4] Thakur M, Loeppert R, Praznik W, Dixit VK (2008) Effect of some ayurvedic vajikarana rasayana herbs on heat induced testicular damage in male rats. Inter J Comp Med 5: 1–14.

[5] Manjunatha G, Tyagi S, Srinivasan K (2004) Safed musli: A White Gold. (*Agrobios*, India).

[6] Acharya D, Mitaine-Offer AC, Kaushik N (2009) Cytotoxic spirostane-type saponins from the roots of *Chlorophytum borivilianum* . J Nat Prod 72: 177–181. 19128156 10.1021/np800559z

[7] Deore SL, Khadabadi SS (2010) Effect of *Chlorophytum borivilianum* on adjuvant induced arthritis in rats. Ann Biol Res 1(1): 36–40.

[8] Haralampidis K, Trojanowska M, Osbourn AE (2002) Biosynthesis of triterpenoid saponins in plants. Adv Biochem Eng Biotechnol 75: 31–49. 11783842 10.1007/3-540-44604-4_2

[9] Rohdich F, Kis K, Bacher A, Eisenreich W (2001) The nonmevalonate pathway of isoprenoids: genes, enzymes and intermediates. Curr Opin Chem Biol 5: 535–540. 11578926 10.1016/s1367-5931(00)00240-4

[10] Rohmer M (2003) Mevalonate-independent methylerythritol phosphate pathway for isoprenoid biosynthesis. Elucidation and distribution. Pure Appl Chem 75: 375–387.

[11] Corey EJ, Matsuda SPT, Bartel B (1993) Isolation of an *Arabidopsis thaliana* gene encoding cycloartenol synthase by functional expression in a yeast mutant lacking lanosterol synthase by the use of a chromatographic screen. Proc Natl Acad Sci USA 90: 11628–11632. 7505443 10.1073/pnas.90.24.11628 PMC48037

[12] Herrera JB, Bartel B, Wilson WK, Matsuda SP (1998) Cloning and characterization of the *Arabidopsis thaliana* lupeol synthase gene. Phytochemistry 49: 1905–11. 9883589 10.1016/s0031-9422(98)00366-5

[13] Kumar S, Kalra S, Kumar S, Kaur J, Singh K (2012) Differentially expressed transcripts from leaf and root tissue of *Chlorophytum borivilianum*: A plant with high medicinal value. Gene 511: 79–87 10.1016/j.gene.2012.09.046 23000016

[14] Kalra S, Kumar S, Lakhanpal N, Kaur J, Singh K (2013) Characterization of Squalene synthase Gene from *Chlorophytum borivilianum* (Sant. and Fernand.). Mol. Biotechnol. DOI 10.1007/s12033-012-9645-1. 23338982

[15] Parkinson J (2009) Expressed Sequence Tags (ESTs) Generation and Analysis, Methods in Molecular Biology. Vol 533. Humana Press, New York.

[16] Rudd S (2003) Expressed sequence tags: alternative or complement to whole genome sequences. Trends Plant Sci 8: 321–329. 12878016 10.1016/S1360-1385(03)00131-6

[17] Morozova O, Hirst M, Marra MA (2009) Applications of new sequencing technologies for transcriptome analysis. Annu Rev Genomics Hum Genet 10: 135–151. 19715439 10.1146/annurev-genom-082908-145957

[18] Shendure J, Ji H (2008) Next-generation DNA sequencing. Nat Biotechnol 26: 1135–1145. 18846087 10.1038/nbt1486

[19] Wang XW, Luan JB, Li JM, Bao YY, Zhang CX, Liu SS (2010) *De novo* characterization of a whitefly transcriptome and analysis of its gene expression during development. BMC Genomics 11: 400. 20573269 10.1186/1471-2164-11-400 PMC2898760

[20] Zerbino DR, Birney E (2008) Velvet: algorithms for de novo short read assembly using de Bruijn graphs. Genome Res 18: 821–829. 18349386 10.1101/gr.074492.107 PMC2336801

[21] Brain KR, Turner TO (1975) The Practical Evaluation of Phytopharmaceuticals. Wright-Scientecknica Bristol, pp 78–80.

[22] Ghawana S, Paul A, Kumar H, Kumar A, Singh H, et al (2011) An RNA isolation system for plant tissues rich in secondary metabolites. BMC Res Notes 4: 85. 21443767 10.1186/1756-0500-4-85 PMC3079660

[23] Schuhr CA, Radykewicz T, Sagner S, Latzel C, Zenk MH, et al (2003) Quantitative assessment of crosstalk between the two isoprenoid biosynthesis pathways in plants by NMR spectroscopy. Phytochem Rev 2: 3–16.

[24] Ashburner M, Ball CA, Blake JA, Botstein D, Butler H, et al (2000) Gene Ontology: tool for the unification of biology. Nature Genetics 25: 25–29. 10802651 10.1038/75556 PMC3037419

[25] Jiang H, Wong WH (2008) SeqMap: mapping massive amount of oligonucleotides to the genome. Bioinformatics 24: 2395–2396. 18697769 10.1093/bioinformatics/btn429 PMC2562015

[26] Mortazavi A, Williams BA, McCue K, Schaeffer L, Wold B (2008) Mapping and quantifying mammalian transcriptomes by RNA-Seq. Nat Methods 5: 621–628. 18516045 10.1038/nmeth.1226 PMC13303166

[27] Pertea G, Huang XQ, Liang F, Antonescu V, Sultana R, et al (2003) TIGR gene indices clustering tools (TGICL): a software system for fast clustering of large EST datasets. Bioinformatics 19: 651–652. 12651724 10.1093/bioinformatics/btg034

[28] Huang XQ, Madan A (1999) CAP3: a DNA sequence assembly program. Genome Research 9: 868–877. 10508846 10.1101/gr.9.9.868 PMC310812

[29] Weber AP, Weber KL, Carr K, Wilkerson C, Ohlrogge JB (2007) Sampling the Arabidopsis transcriptome with massively parallel pyrosequencing. Plant Physiol 144: 32–42. 17351049 10.1104/pp.107.096677 PMC1913805

[30] Mane SP, Evans C, Cooper KL, Crasta OR, Folkerts O, et al (2009) Transcriptome sequencing of the Microarray Quality Control (MAQC) RNA reference samples using next generation sequencing. BMC Genomics 10: 264. 19523228 10.1186/1471-2164-10-264 PMC2707382

[31] Gahlan P, Singh HR, Shankar R, Sharma N, Kumari A, et al (2012) De novo sequencing and characterization of Picrorhiza kurrooa transcriptome at two temperatures showed major transcriptome adjustments. BMC Genomics 13: 126. 22462805 10.1186/1471-2164-13-126 PMC3378455

[32] Clark SE, Williams RW, Meyerowitz EM (1997) The CLAVATA1 gene encodes a putative receptor kinase that controls shoot and floral meristem size in Arabidopsis. Cell 89(4): 575–85. 9160749 10.1016/s0092-8674(00)80239-1

[33] Hirayama T, Oka A (1992) Novel protein kinase of Arabidopsis thaliana (APK1) that phosphorylates tyrosine, serine and threonine. Plant Mol Biol 20(4): 653–62. 1450380 10.1007/BF00046450

[34] Yanhui C, Xiaoyuan Y, Kun H, Meihua L, Jigang L, et al (2006) The MYB transcription factor superfamily of Arabidopsis: expression analysis and phylogenetic comparison with the rice MYB family. Plant Mol Biol 60(1): 107–124. 16463103 10.1007/s11103-005-2910-y

[35] Singh K, Foley RC, Onate-Sanchez L (2002) Transcription factors in plant defense and stress responses. Curr Opin Plant Biol 5(5): 430–436. 12183182 10.1016/s1369-5266(02)00289-3

[36] Li Z, Thomas TL (1998) PEI1, an embryo-specific zinc finger protein gene required for heart-stage embryo formation in Arabidopsis. Plant Cell 10: 383–398. 9501112 10.1105/tpc.10.3.383 PMC143998

[37] Sung S, Schmitz RJ, Amasino RM (2006) A PHD finger protein involved in both the vernalization and photoperiod pathways in Arabidopsis. Genes Dev 20(23): 3244–8. 17114575 10.1101/gad.1493306 PMC1686601

[38] Heim MA, Jakoby M, Werber M, Martin C, Weisshaar B, et al (2003) The basic helix-loop-helix transcription factor family in plants: a genome-wide study of protein structure and functional diversity. Mol Biol Evol 20: 735–747. 12679534 10.1093/molbev/msg088

[39] Carels N, Hatey P, Jabbari K, Bernardi G (1998) Compositional properties of homologous coding sequences from plants. J Mol Evol 46: 45–53. 9419224 10.1007/pl00006282

[40] Wei W, Qi X, Wang L, Zhang Y, Hua W, et al (2011) Characterization of the sesame (*Sesamum indicum L.*) global transcriptome using Illumina paired-end sequencing and development of EST-SSR markers. BMC Genomics 12: 451. 21929789 10.1186/1471-2164-12-451 PMC3184296

[41] Geetha KA, Maiti S (2001) Reproductive biology of Safed musli (*Chlorophytum borivilianum* Sant. & Fern.). Journal of Tropical Medicinal Plants. 2(2): 287–290.

[42] Senthilvel S, Jayashree B, Mahalakshmi V, Kumar PS, Nakka S, et al (2008) Development and mapping of simple sequence repeat markers for pearl millet from data mining of expressed sequence tags. BMC Plant Biol 8: 119. 19038016 10.1186/1471-2229-8-119 PMC2632669

[43] Varshney RK, Thiel T, Stein N, Langridge P, Graner A (2002) *In silico* analysis on frequency and distribution of microsatellites in ESTs of some cereal species. Cell Mol Biol Lett 7: 537–546. 12378259

[44] Visavadiya NP, Soni B, Dalwadi N, Madamwar D (2010) *Chlorophytum borivilianum* as potential terminator of free radicals in various in vitro oxidation systems. Drug Chem Toxicol 33(2) 173–82 10.3109/01480540903311068.47 20307144

[45] Wise ML, Croteau R (1998): Monoterpene biosynthesis. In Comprehensive natural products chemistry. Volume 2. Edited by: Cane DE. Oxford: Pergamon Press.

[46] Schuhr CA, Radykewicz T, Sagner S, Latzel C, Zenk MH, et al (2003) Quantitative assessment of crosstalk between the two isoprenoid biosynthesis pathways in plants by NMR spectroscopy. Phytochem Rev 2: 3–16.

[47] Qureshi N, Porter W (1981): Conversion of acetyl-coenzyme A to isopentenyl pyrophosphate. In Biosynthesis of Isoprenoid Compounds. Volume 1. Edited by: Porter JW, Spurgeon SL. New York: John Wiley.

[48] Eisenreich W, Schwarz M, Cartayrade A, Arigoni D, Zenk MH, et al (1998) The deoxyxylulose phosphate pathway of terpenoid biosynthesis in plants and microorganisms. Chem Biol 5: 221–233. 10.1016/s1074-5521(98)90002-3 9751645

[49] Duvold T, Bravo JM, Pale GC, Rhomer M (1997) Biosynthesis of 2-C-methyl-D- erythritol, a putative C-5 intermediate in the mevalonate independent pathway for isoprenoid biosynthesis. Tetrahedron Lett 38: 4769–4772.

[50] Park J, Rhee D, Lee Y (2005) Biological activities and chemistry of saponins from *Panax ginseng* C. A. Meyer. Phytochemistry Rev 4(2): 159–175.

[51] Ohyama K, Suzuki M, Kikuchi J, Saito K, Muranaka T (2009) Dual biosynthetic pathways to phytosterol via cycloartenol and lanosterol in Arabidopsis. Proc Natl Acad Sci USA 106(3): 725–730. 19139393 10.1073/pnas.0807675106 PMC2621255

[52] Kushiro T, Shibuya M, Ebizuka Y (1998) b-Amyrin synthase: cloning of oxidosqualene cyclase that catalyzes the formation of the most popular triterpene among higher plants. Eur J Biochem 256: 238–244. 9746369 10.1046/j.1432-1327.1998.2560238.x

[53] Tansakul P, Shibuya M, Kushiro T, Ebizuka Y (2006) Dammarenediol-II synthase, the first dedicated enzyme for ginsenoside biosynthesis, in *Panax ginseng* . FEBS Lett 580(22): 5143–5149. 16962103 10.1016/j.febslet.2006.08.044

[54] Takeuchi A, Matsumoto S, Hayatsu M (1994) Chalcone synthase from Camellia sinensis: isolation of the cDNAs and the organ-specific and sugar responsive expression of the genes. Plant Cell Physiol 35: 1011–1018. 7820373

[55] Lin GZ, Lian YJ, Ryu JH, Sung MK, Park JS, et al (2007) Expression and purification of His-tagged flavonol synthase of Camellia sinensis from Escherichia coli. Protein Exp Puri 55: 287–292. 10.1016/j.pep.2007.05.013 17629496

[56] Kreuzaler F, Ragg H, Fautz E, Kuhn DN, Hahlbrock K (1983) UV-induction of chalcone synthase mRNA in cell suspension cultures of Petroselinum hortense. Proc Natl Acad Sci USA 80: 2591–2593. 16593307 10.1073/pnas.80.9.2591 PMC393872

[57] Goff SA, Klein TM, Roth BA, Fromm ME, Cone KC, et al (1990) Transactivation of anthocyanin biosynthetic genes following transfer of B regulatory genes into maize tissues. EMBO J 9: 2517–2522. 2369901 10.1002/j.1460-2075.1990.tb07431.x PMC552281

[58] Shirley BW, Hanley S, Goodman H (1992) Effects of ionizing radiation on a plant genome: analysis of two Arabidopsis *transparent testa* mutations. Plant Cell 4: 333–347. 1354004 10.1105/tpc.4.3.333 PMC160133

[59] Boss PK, Davies C (1996a) RobinsonS (1996a) Analysis of the expression of anthocyanin pathway genes in developing *Vitis vinifera* L. cv. 'Shiraz' grape berries and the implications for pathway regulation. Plant Physiol 111: 1059–1066. 12226348 10.1104/pp.111.4.1059 PMC160981

[60] Moriguchi T, Kita M, Ogawa K, Tomono Y, Endo T, et al (2002) Flavonol synthase gene expression during citrus fruit development. Physiologica Plantarum 114: 251–258. 10.1034/j.1399-3054.2002.1140211.x 11903972

[61] Manning K (1998) Isolation of a set of ripening-related genes from strawberry: their identification and possible relationship to fruit quality traits. Planta 205: 622–631. 9684364 10.1007/s004250050365

[62] Yu O, McGonigle B (2005) Metabolic Engineering of Isoflavone Biosynthesis. Adv Agronomy 86: 147–190.

[63] Meijer AH, Souer E, Verpoorte R, Hoge JHC (1993) Isolation of cytochrome P450 cDNA clones from the higher plant *Catharanthus roseus* by a PCR strategy. Plant Mol Biol 22: 379–383. 8507838 10.1007/BF00014944

[64] Bak S, Kahn RA, Olsen CE, Halkier BA (1997) Cloning and expression in Escherichia coli of the obtusifoliol 14 alpha-demethylase of *Sorghum* bicolor (L.) Moench, a cytochrome P450 orthologous to the sterol 14 alpha-demethylases (CYP51) from fungi and mammals. Plant J 11: 191–201. 9076987 10.1046/j.1365-313x.1997.11020191.x

[65] Cabello-Hurtado F, Zimmerlin A, Rahier A, Taton M, DeRose R, et al (1997) Cloning and functional expression in yeast of a cDNA coding for an obtusifoliol 14α- demethylase (CYP51) in wheat. Biophys Biochem Res Commun 230: 381–385. 10.1006/bbrc.1996.5873 9016788

[66] Morikawa T, Mizutani M, Aoki N, Watanabe B, Saga H, et al (2006) Cytochrome P450 CYP710A encodes the sterol C-22 desaturase in Arabidopsis and tomato. Plant Cell 18: 1008–1022. 16531502 10.1105/tpc.105.036012 PMC1425849

[67] Bowles D et al (2006) Glycosyltransferases of lipophilic small molecules. Annu Rev Plant Biol 57: 567–597. 16669774 10.1146/annurev.arplant.57.032905.105429

[68] Shibuya M, Nishimura K, Yasuyama N, Ebizuka Y (2010) Identification and characterization of glycosyltransferases involved in the biosynthesis of soyasaponin I in *Glycine max* . FEBS Lett 584: 2258–2264. 20350545 10.1016/j.febslet.2010.03.037

[69] Caporale LH (1995) Chemical ecology: a view from the pharmaceutical industry. Proc Natl Acad Sci USA 92: 75–82. 7816850 10.1073/pnas.92.1.75 PMC42819

[70] Rueffer M, Zenk MH (1987) Distant precursors of benzylisoquinoline alkaloids and their enzymatic formation. Z. Naturforsch 42C: 319–32.

[71] Marques IA, Brodelius PE (1988) Elicitorinduced L-tyrosine decarboxylase from plant cell suspension cultures: I. Induction and purification. Plant Physiol 88: 47–51. 10.1104/pp.88.1.46 PMC1055523 16666277

[72] Facchini PJ, De Luca V (1994) Differential and tissue-specific expression of a gene family for tyrosine/dopa decarboxylase in opium poppy. J Biol Chem 269: 26684–90. 7929401

[73] Maldonado-Mendoza IE, L'opez-Meyer M, Galef JR, Burnett RJ, Nessler CL (1996) Molecular analysis of a new member of the opium poppy tyrosine/3,4- dihydroxyphenylalanine decarboxylase gene family. Plant Physiol 110: 43–49. 8587993 10.1104/pp.110.1.43 PMC157692

[74] Kawalleck P, Keller H, Hahlbrock K, Scheel D, Somssich IE (1993) A pathogen responsive gene of parsley encodes tyrosine decarboxylase. J Biol Chem 268: 2189–94. 8420986

[75] Trezzini GF, Horrichs A, Sommssich IE (1993) Isolation of putative defenserelated genes from *Arabidopsis thaliana* and expression in fungal elicitor-treated cells. Plant Mol Biol 21: 385–89. 8425063 10.1007/BF00019954

[76] Schmelzer E, Kr¨uger-Lebus S, Hahlbrock K (1989) Temporal and spatial patterns of gene expression around sites of attempted fungal infection in parsley leaves. Plant Cell 1: 993–1001. 12359883 10.1105/tpc.1.10.993 PMC159836

[77] Stadler R, Kutchan TM, Loeffler S, Nagakura N, Cassels B, et al (1987) Revision of the early steps of reticuline biosynthesis. Tetrahedron Lett 28: 1251–54.

[78] Sato F, Tsujita T, Katagiri Y, Yoshida S, Yamada Y (1994) Purification and characterization of *S*-adenosyl-L-methionine: noncoclaurine 6-*O*-methyltransferase from cultured *Coptis japonica* cells. Eur J Biochem 225: 125–31. 7925429 10.1111/j.1432-1033.1994.00125.x

[79] Morishige T, Tsujita T, Yamada Y, Sato F (2000) Molecular characterization of the *S*-adenosyl-L-methionine: 30-hydroxy- *N*-methylcoclaurine-40-*O*-methyltransferase of isoquinoline alkaloid biosynthesis in *Coptis japonica* . J Biol Chem 275: 23398–405. 10811648 10.1074/jbc.M002439200

[80] Kumar M, Meena P, Verma S, Kumar M, Kumar A (2010) Anti-tumour, Anti-mutagenic and Chemomodulatory Potential of *Chlorophytum borivilianum.* . A Pac Jour Canc Preven 11: 327–334. 20843110

[81] Qiu SX, Li XC, Xiong Y, Dong Y, Chai H, et al (2000) Isolation and characterization of cytotoxic saponin Chloromaloside-A from *Chlorophytum* . Planta Med 66: 587–90. 10985095 10.1055/s-2000-8600

[82] Li Q, Wang X, Yang Z, Wang B, Li S (2009) Menthol induces cell death via the TRPM8 channel in the human bladder cancer cell line T24. Oncology 77(6): 335–41. 19955836 10.1159/000264627

