# Supplementary figures and images for: De Novo Transcriptome Sequencing Reveals Important Molecular Networks and Metabolic Pathways of the Plant, Chlorophytum borivilianum
Source: PLoS One. 2013 Dec 23;8(12):e83336. doi: 10.1371/journal.pone.0083336 (PMC3871651; doi:10.1371/journal.pone.0083336)

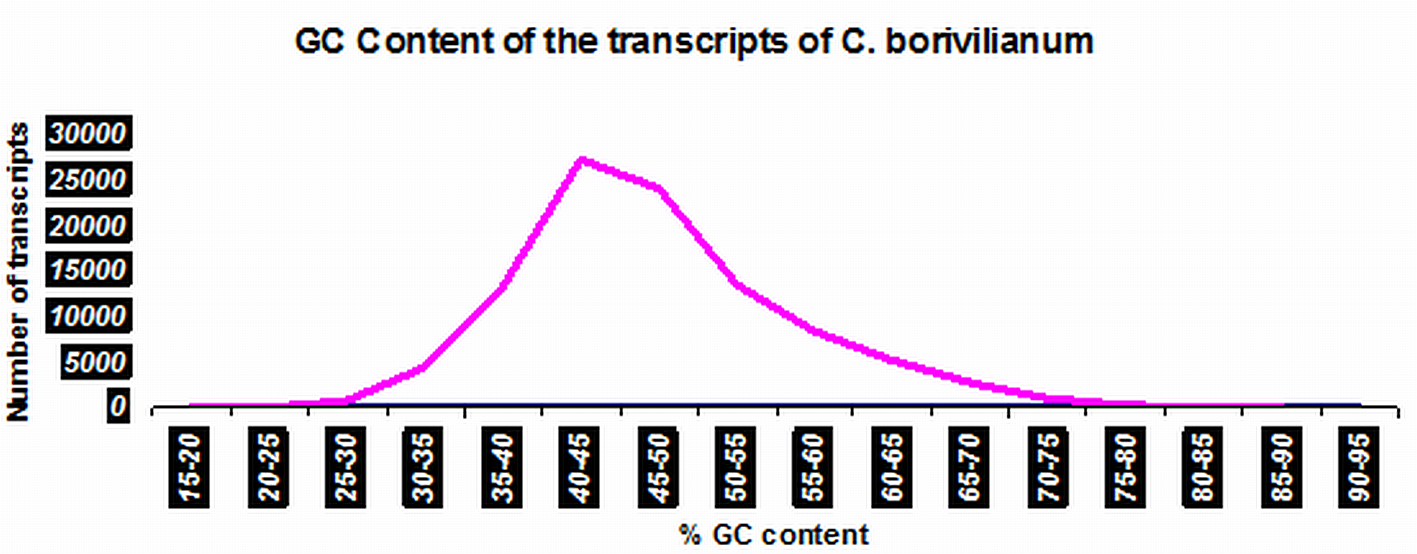

Supplement: Figure S1 — Guanine-cytosine (GC) content analysis of C. borivilianum transcripts. (TIF) (TIF) [file pone.0083336.s001.tif]

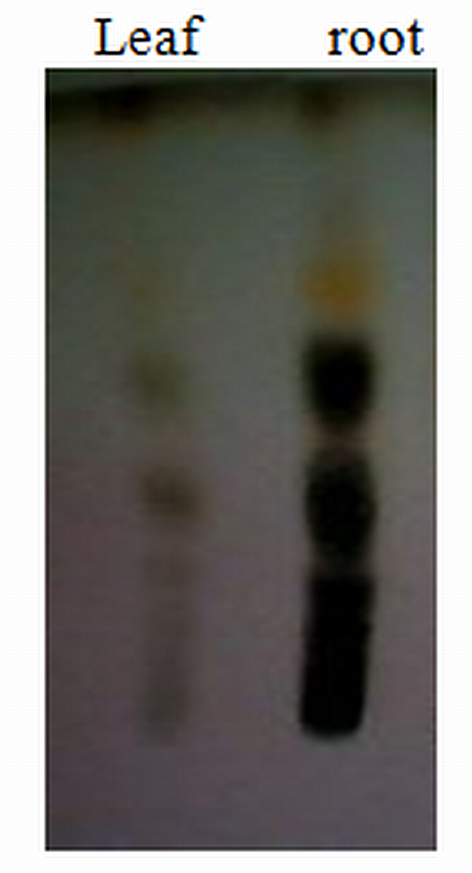

Supplement: Figure S2 — Analysis of total saponins contents in root and leaf tissue of C borivilianum by soxhelation method and TLC. (TIF) [file pone.0083336.s002.tif]

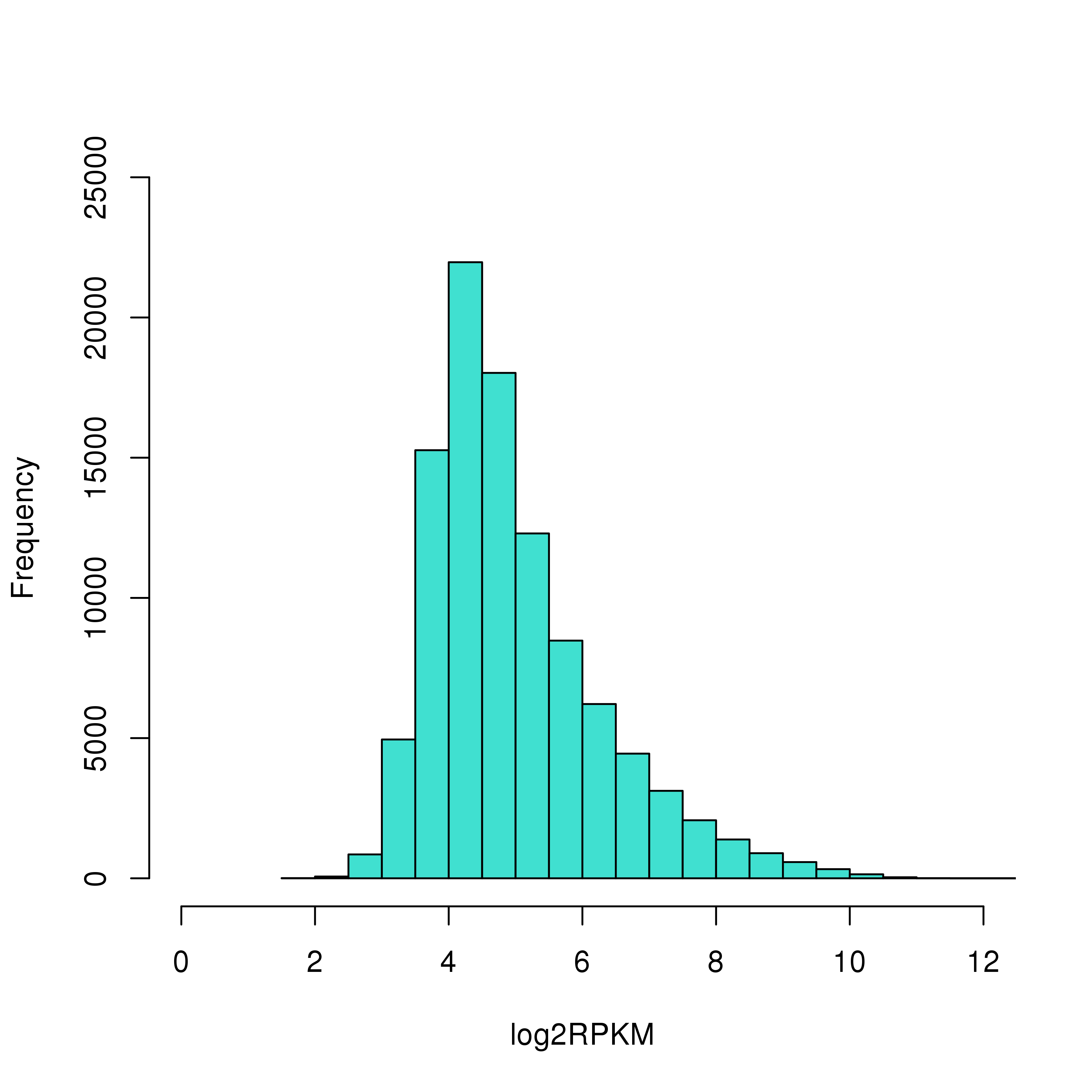

Supplement: Figure S3 — Distribution of RPKM values for different transcripts in C. borivilianum transcriptome. (TIF) [file pone.0083336.s003.tif]
