# Supplementary material for: De Novo Transcriptome Sequencing Reveals Important Molecular Networks and Metabolic Pathways of the Plant, Chlorophytum borivilianum
Source: PLoS One. 2013 Dec 23;8(12):e83336. doi: 10.1371/journal.pone.0083336 (PMC3871651; doi:10.1371/journal.pone.0083336)
Supplement: Table S4 — Details on transcription factor (TF) families identified in C. borivilianum. (XLS) [file pone.0083336.s007.xls]

| **S.No** | **Transcription factor family name** | **Number** |
| --- | --- | --- |
| 1 | C3H | 1301 |
| 2 | PHD | 1142 |
| 3 | MYB | 1031 |
| 4 | FAR1 | 813 |
| 5 | MYB-related | 754 |
| 6 | bHLH | 700 |
| 7 | MADS | 669 |
| 8 | C2H2 | 526 |
| 9 | Orphans | 514 |
| 10 | SET | 496 |
| 11 | SNF2 | 473 |
| 12 | TRAF | 462 |
| 13 | mTERF | 436 |
| 14 | WRKY | 427 |
| 15 | HB | 392 |
| 16 | NAC | 383 |
| 17 | AP2-EREBP | 338 |
| 18 | FHA | 281 |
| 19 | ABI3VP1 | 228 |
| 20 | bZIP | 208 |
| 21 | AUX/IAA | 199 |
| 22 | GNAT | 156 |
| 23 | Jumonji | 144 |
| 24 | Tify | 118 |
| 25 | G2-like | 114 |
| 26 | Trihelix | 113 |
| 27 | LUG | 108 |
| 28 | DDT | 107 |
| 29 | SBP | 98 |
| 30 | GRAS | 90 |
| 31 | ARF | 88 |
| 32 | ARID | 85 |
| 33 | DBP | 78 |
| 34 | CCAAT | 74 |
| 35 | HMG | 71 |
| 36 | TCP | 67 |
| 37 | CSD | 66 |
| 38 | LOB | 64 |
| 39 | C2C2-CO-like | 60 |
| 40 | Pseudo ARR-B | 56 |
| 41 | BSD | 45 |
| 42 | SWI/SNF-BAF60b | 44 |
| 43 | TUB | 42 |
| 44 | EIL | 42 |
| 45 | HSF | 39 |
| 46 | TAZ | 37 |
| 47 | PBF-2-like | 34 |
| 48 | RWP-RK | 34 |
| 49 | E2F-DP | 33 |
| 50 | CPP | 29 |
| 51 | LIM | 24 |
| 52 | BES1 | 23 |
| 53 | OFP | 23 |
| 54 | PLATZ | 22 |
| 55 | Alfin-like | 19 |
| 56 | BBR/BPC | 16 |
| 57 | C2C2-YABBY | 14 |
| 58 | zf-HD | 13 |
| 59 | GeBP | 10 |
| 60 | MBF1 | 7 |
| 61 | NOZZLE | 4 |
| 62 | IWS1 | 3 |
